# Weight shapes the intestinal microbiome in preterm infants: results of a prospective observational study

**DOI:** 10.1186/s12866-021-02279-y

**Published:** 2021-07-21

**Authors:** Fardou H. Heida, Elisabeth M. W. Kooi, Josef Wagner, Thi-Yen Nguyen, Jan B. F. Hulscher, Anne G. J. F. van Zoonen, Arend F. Bos, Hermie J. M. Harmsen, Marcus C. de Goffau

**Affiliations:** 1 Division of Obstetrics & Gynecology, Isala Klinieken, University of Groningen, Zwolle, the Netherlands; 2 Division of Pediatric Surgery Beatrix Children’s Hospital, University Medical Center Groningen, University of Groningen, Groningen, the Netherlands; 3 Division of Neonatology Beatrix Children’s Hospital, University of Groningen, University Medical Center Groningen, Groningen, the Netherlands; 4 Victorian Infectious Diseases Reference Laboratory, Peter Doherty Institute for Infection and Immunity, Royal Melbourne Hospital, Melbourne, Australia; 5 Division of Microbiology, University of Groningen, University Medical Center Groningen, Groningen, the Netherlands; 6 Department of Vascular Medicine, Academic Medical Center, University of Amsterdam, Amsterdam, the Netherlands; 7 Parasites and Microboes, Wellcome Sanger Institute, Wellcome Genome Campus, Hinxton, Cambridge, United Kingdom

**Keywords:** Intestinal microbiome, Prematurity, Mode of delivery, Development, Weight

## Abstract

**Background:**

The intestinal microbiome in preterm infants differs markedly from term infants. It is unclear whether the microbiome develops over time according to infant specific factors.

**Methods:**

We analysed (clinical) metadata - to identify the main factors influencing the microbiome composition development - and the first meconium and faecal samples til the 4th week via 16 S rRNA amplican sequencing.

**Results:**

We included 41 infants (gestational age 25–30 weeks; birth weight 430-990 g. Birth via Caesarean section (CS) was associated with placental insufficiency during pregnancy and lower BW. In meconium samples and in samples from weeks 2 and 3 the abundance of *Escherichia* and *Bacteroides* (maternal faecal representatives) were associated with vaginal delivery while *Staphylococcus* (skin microbiome representative) was associated with CS. Secondly, irrespective of the week of sampling or the mode of birth, a transition was observed as children children gradually increased in weight from a microbiome dominated by *Staphylococcus* (Bacilli) towards a microbiome dominated by *Enterobacteriaceae* (Gammaproteobacteria).

**Conclusions:**

Our data show that the mode of delivery affects the meconium microbiome composition. They also suggest that the weight of the infant at the time of sampling is a better predictor for the stage of progression of the intestinal microbiome development/maturation than postconceptional age as it less confounded by various infant-specific factors.

**Supplementary Information:**

The online version contains supplementary material available at 10.1186/s12866-021-02279-y.

## Introduction

Extreme prematurity involves high mortality and morbidity [1]. Diseases such as necrotizing enterocolitis (NEC) are related to prematurity with aberrant gut microbiome colonization patterns [2–4]. For example, NEC has been associated with a particular group of clostridial species closely related to *Clostridium perfringens* in many studies, while staphylococci appear to be associated with a decreased risk for NEC development [5–13].

Previous studies on the intestinal microbiome of preterm infants, using 16 S rRNA-based sequencing technologies, have revealed remarkable differences with the microbiome of term infants, including higher abundances of bacilli and Gammaproteobacteria [14–26]. The process of microbiome maturation is important in understanding differences between the microbiomes of preterm- and term infants. In preterm infants, the prolonged absence of bacteria that usually colonize and protect a term infants (bifidobacteria) is a clear indicator that the intestinal maturation process is either severely disturbed or altered [5, 6, 21, 26–29].

Besides host biology, such as low birth weight (BW) and immaturity of organs including the gastrointestinal tract as a result of low gestational age (GA), there are multiple other exogenous factors that could affect the intestinal microbiome maturation of preterm infants (i.e. mode of delivery, neonatal feeding regime, the neonatal intensive care unit (NICU) environment and peri- and postnatal antibiotic exposure) [5, 6, 14, 16, 19, 29].

While interest in this subject is growing, the focus of research is typically, with a few exceptions [15–19, 23], on the relation between the intestinal microbiome of the preterm infant and disease instead of on the development of the microbiome composition over time. We aimed to determine whether the early microbiome development in extremely and very premature infants born between 25 and 30 weeks of gestation is dependant by infant maturation or whether it is mainly characterized by varying states of dysbiosis. Secondly, we aimed to analyse whether exogenous factors were intricately linked with microbiome maturation.

## Results

### Patient characteristics

**Fig. 1:**
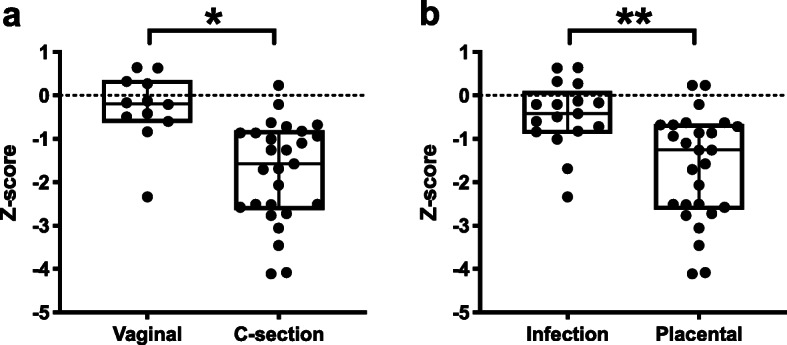
Boxplot of the relation between birthweight z-scores and the **a** mode of delivery and **b** the underlying cause of prematurity. The Y-axis represents the standard deviation in SD units from the Dutch reference growth curves for gestational age and gender. A z-score of 0 represents the P50, which is exactly the age/sex-appropriate median. Interquartile range is shown. * *p* = 0.00008, ** *p* = 0.0005

In this study 41 preterm born infants were included. The GA ranged between 25 and 30 weeks (median 27.6 weeks IQR 26.0-28.1). Table 1 summarizes the baseline characteristics. Detailed patient characteristics per week are shown in Table 2. Important for understanding this cohort, the mode of delivery was strongly associated with the birth weight Z-score for GA and gender (Fig. 1a). Infants delivered by CS had a higher GA on average than their vaginally born counterparts (median 28.1 IQR 26.7–28.2) vs. 26.0 IQR 25.3–26.9 weeks, *p* < 0.01), yet were of lower BW (median 778 IQR 641–923 vs. 835 IQR 800–980 g, *p* = 0.03). Underlying causes of prematurity for all vaginally delivered infants (13/13) were intra-uterine infection, (prolonged) premature rupture of membranes and/or cervix insufficiency while most infants delivered by CS were born preterm because of placental insufficiency (22/27) (Table 1). Placental insufficiency was negatively associated with the Z-score for BW (*p* < 0.01, Fig. 1b).

**Table 1:** Baseline characteristics

	*n* = 41
**Male gender (%)**	17 (42 %)
**Gestational age, wk (range)**	27.6 (25–30)
**Birth weight, g (range)**	820 (430–990)
**Caesarean section (%)**	27 (66 %)
** - Infection/spontaneous contractions**	5 (19 %)
** - Placental insufficiency**	22 (81 %)
**NEC development (maximal Bell’s stage)**	6 (15 %)
**Mortality before discharge (%)**	8 (20 %)
**Cause of death (%)**	
**NEC**	4 (50 %)
**Respiratory failure**	4 (50 %)

**Table 2:** Patient characteristics in the four weeks postpartum

	Week 1 *n* = 31	Week 2 *n* = 37	Week 3 *n* = 35	Week 4 *n* = 32
**Corrected age in days (range)**	4 (1–6)	8 (8–11)	15 (15–19)	22 (22–29)
**Weight, g (range)**	831 (495–1040)	864 (483–1230)	969 (570–1235)	1095 (705–1675)
**Mothers’ milk usage (%)**	16 (40)	34 (87)	25 (69)	21 (66)
**Antibiotic exposure at same day as faeces sample day (%)**	26 (65)	12 (31)	17 (41)	5 (16)

### Intestinal microbiome development over time

We analyzed 142 samples (3.5 samples per patient on average, range 2–4). These samples included one faecal sample per week, starting with the first meconium (after birth), and afterwards the first faecal sample of every week during the first four weeks after birth.

**Fig. 2:**
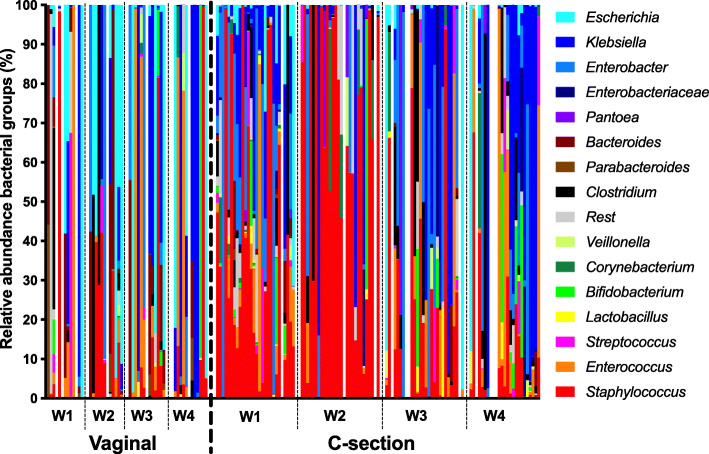
Overview relative abundance main bacterial groups per week (W1-4) in preterm infants delivered vaginally or by CS. Bacterial groups are listed on the genus level with the exception of all *Enterobacteriaceae* species that were not *Escherichia*, *Klebsiella* or *Enterobacter*. The samples were sorted based upon their patient number in a paired fashion so samples can be tracked over time. All *Enterobacteriaceae* genera have blue hues for visualization purposes

An overview of the abundances (% of reads per sample) of the most important bacterial groups (species/genera) in these 4 timepoints is shown in Fig. 2 and exact details of oligotype abundances are given in Supplementary Information file 1. Within meconium samples the *Staphylococcus* genus, mostly consisting out of *S. epidermidis*, was frequently the most dominant bacterial group (40 % of reads on average) followed by 4 members from the *Enterobacteriaceae* family; *Enterobacter cloacae/hormaechei* (11 %), *Escherichia/Shigella* (10 %), *Klebsiella oxytoca/michiganensis* (5.9 %) and *Klebsiella pneumoniae/variicola* (5.7 %). In week 2 an increase in the abundance of *Staphylococcus* was observed in this cohort (50 % of reads on average). A decline however of *Staphylococcus* is observed in weeks 3 and 4 (17 and 11 %, respectively) with the *Enterobacteriaceae* family becoming dominant in most samples (32 %, 27 %, 52 and 54 % on average for weeks 1, 2, 3 and 4, respectively). Bifidobacteria and lactobacilli were groups of very minor importance within this cohort. Whilst several of the main species such as *S. epidermidis*, *K. oxytoca/michiganensis* & *K. pneumonia/variicola* can be identified down to the species level using V4 16 S rRNA amplicon sequencing to a reasonable degree this is not the case for *Escherichia coli* as methods other than 16 S rRNA amplicon sequencing are required to tell the genera *Escherichia* and *Shigella* apart. However, from both culture [30] and whole genome shotgun sequencing [31] the identities of the most abundant species in this study seem relatively certain, including *E. coli*. Shigellosis rarely affects infants during the first month of life whilst infections with *E. coli* are common [32]. *Escherichia*/*Shigella* reads are hence referred to as *Escherichia*. For purposes of clarity and avoiding unsubstantiated taxonomic depth representation most other bacterial groups have been merged together at the genus level in most analyses. Detailed information on abundances per bacterial group per sample, including representative sequences & metadata are provided in Supplementary Information File 2.

### Mode of delivery and intestinal microbiota development

**Fig. 3:**
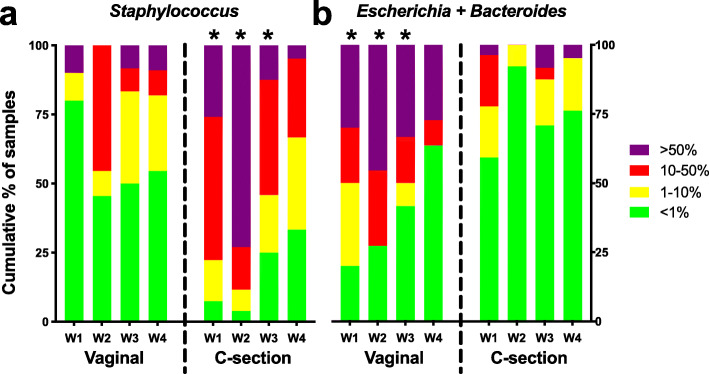
Prolonged effect of the mode of delivery on the faecal microbiota composition during the first weeks of life of preterm infants. **a** The prevalence of *Staphylococcus*, a typical abundant representative of the skin microbiota, is significantly associated with C-section delivery in samples from the first 3 weeks of life. **b** The combined prevalence of *Escherichia coli* and members of the *Bacteroides* genus, typical representatives of the maternal faecal microbiota, is associated significantly with vaginal delivery in samples from the first 3 weeks of life. Blocks represent categories of samples that are from the same week (W1-4) with the same mode of delivery. Prevalence brackets, as shown on the right, represent the total % of reads per sample. For example, more than 75 % of all samples from vaginally delivered infants from week 1 had less than 1 % *Staphylococcus*. Significant differences (*p* < 0.05) between samples of the same week but from different delivery methods are indicated with an asterisk (*) above the category with the highest prevalence

One of the most striking patterns in the data, alluded to in Fig. 2, is that the mode of delivery has a significant influence on the microbiome composition. This was most evident within the first three weeks after birth (Fig. 3). Abundance of *Staphylococcus*, a genus of typical skin bacteria, was significantly associated with CS delivery in samples from the first 3 weeks of life (*p* < 0.01, *p* < 0.01, *p* < 0.05 and *p* = 0.28 for weeks 1–4, respectively). On the other side of the coin *Escherichia* and *Bacteroides* were the most strongly correlated with vaginal delivery. The combined prevalence the facultative anaerobe *Escherichia* and members of the aero-tolerant anaerobic *Bacteroides* genus, typical maternal faecal representatives, was significantly associated with vaginal delivery during these first 3 weeks (*p* = 0.02, *p* < 0.01, *p* = 0.03 and *p* = 1 for weeks 1–4, respectively).

### Mode of delivery and infant weight development

In this cohort the mode of delivery was significantly associated with BW and GA (Fig. 1). The association between the infant’s current weight (not corrected for GA) at sampling time and the mode of delivery remains largely unchanged during the first four weeks (*p* = 0.04, *p* = 0.03, *p* = 0.02 and *p* = 0.04 for weeks 1–4, respectively), as no difference was present in weight gain in g/week of infants delivered vaginally and by CS (*p* = 0.9; *p* = 0.8; *p* = 0.6 for weeks 2–4, respectively). Z-scores from BW were strongly correlated with absolute weight in all 4 weeks (Spearman ρ correlation coefficients of 0.70, 0.67, 0.66 and 0.52, respectively).

### Infant weight and intestinal microbiome development over time

**Fig. 4:**
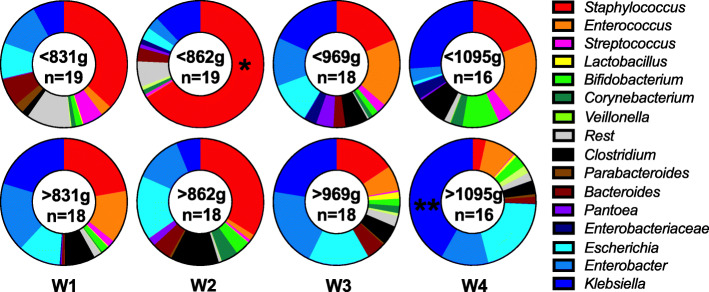
Average abundance of bacterial genera in samples below or above the median weight of each week. Significant differences are found in the bacterial composition of samples from week 2 in regards to the abundance of *Staphylococcus* (*) and in week 4 (**) in regards to the combined *Enterobacteriaceae* abundance (all blue hues)

While the mode of delivery was the most important determinant for the infants’ initial microbiome (Figs. 2 and 3), the increase of *Enterobacteriaceae* in weeks 3 and 4 appeared associated with absolute weight at sampling time (Fig. 4). When comparing infants with an above-median weight (≥835 g) with their lighter counterparts, little difference was observed in the microbiome composition of the meconium (week 1). In week 2, infants with an above median weight (≥860 g) contained significantly less *Staphylococcus* (*p* < 0.01). This particular association was however partially indirect, as infants delivered by CS had a lower median BW and were more frequently colonized during delivery by *Staphylococcus* (Fig. 3a). The weight of infants increased from a mean of 860 g in week 2 to 969 g in week 3 to 1095 g in week 4, respectively. In week 3 *Enterobacteriaceae* became more dominant again at the expense of *Staphylococcus*, as the influence of the mode of delivery declined. In week 4 the correlation coefficient between the abundance of *Enterobacteriaceae* and current weight was significant (ρ = 0.4, *p* = 0.04). More specifically, at a body weight of > 1100 g nearly all samples were dominated by *Enterobacteriaceae*. In general, the absolute weight of infants, irrespective of the week of sample collection, appeared to be (independently) associated with the shift from a *Staphylococcus* dominated gut towards one dominated by *Enterobacteriaceae*.

### Gut microbiome maturation vs. infant weight or postconceptional age

**Fig. 5:**
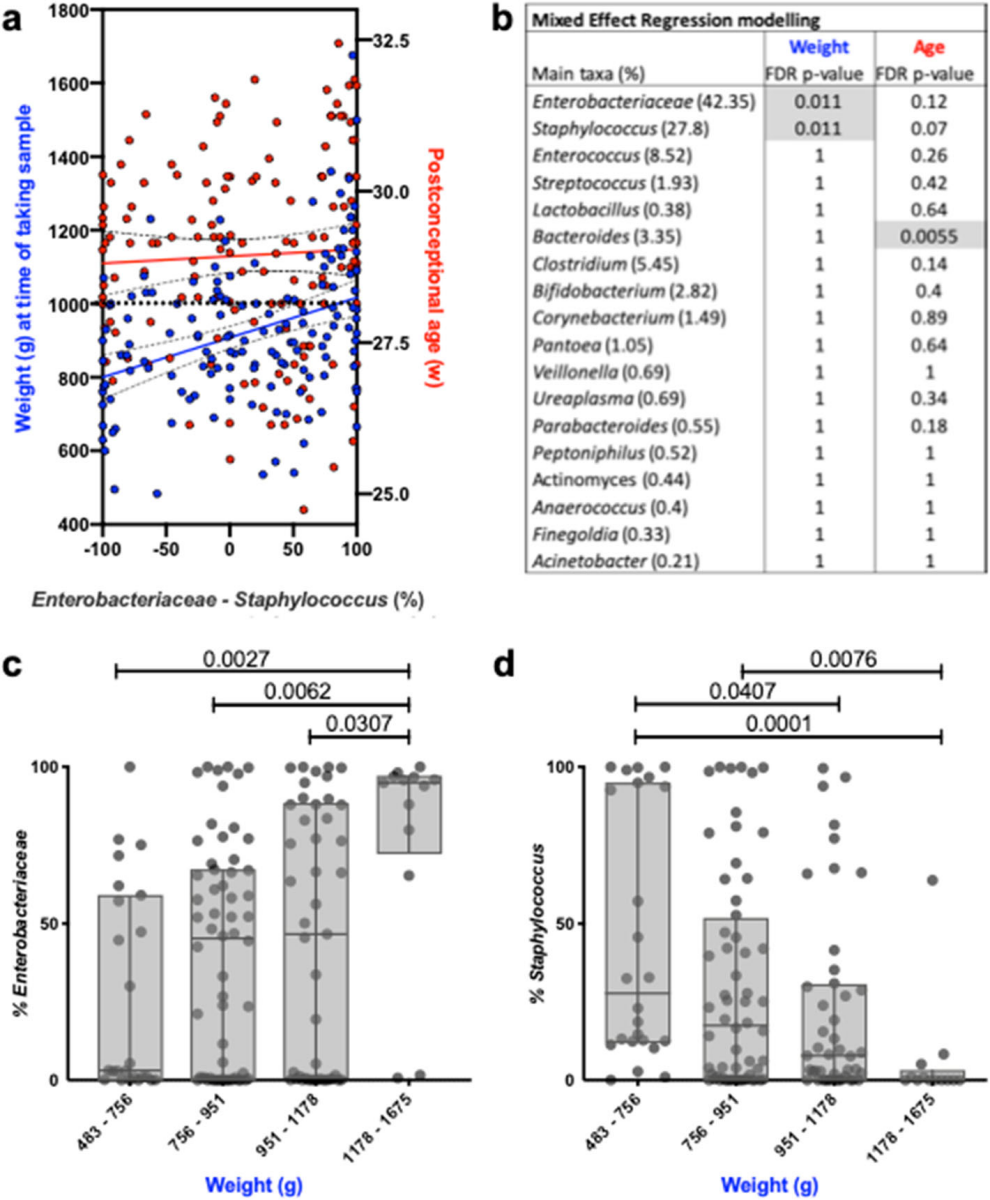
Regression analyses of gut microbiota development and host maturity markers. **a** All 142 samples from all 4 timepoints were used for a linear regression analyses with the result of the difference between *Enterobacteriaceae* and *Staphylococcus* (%) on the X-axis and either the infant weight measured in grams at the time of taking the sample on the left-Y-axis (blue) or the postconceptional age of an infant at the time of taking a sample on the right-Y-axis (red). The slope of regression analysis using weight (blue) was significantly non-zero (Y = 1.08X + 908, *p* < 0.0001) while the slope of the regression analysis using postconceptional age was not significantly non-zero (Y = 0.0012X + 28.92, *p* = 0.57). **b** A mixed effect regression modeling analysis of the main taxonomic groups of importance was performed to take the longitudinal and paired aspect of this dataset into account. This analysis shows that the abundance of *Enterobacteriaceae* and *Staphylococcus* are both significantly discriminating features in regards to weight, in opposite directions, but that they are not significantly associated with postconceptional age. *Bacteroides* was found to be discriminating feature in regards to postconceptional age but this is due to the association of *Bacteroides* with vaginal delivery, and the fact that vaginally delivered infants had a lower postconceptional age when born yet had a higher birthweight, on average, due to the underlying causes of prematurity. **c **&** d** When *Enterobacteriaceae* and *Staphylococcus* were plotted in four weight groups, which were determined from the mixed effect regression modelling, significant differences were found between the different weight groups using the non-parametric Kruskal-Wallis test with Dunn’s multiple comparison test. A FDR corrected p value is shown, when below 0.05. Interquartile ranges are shown

A simple linear regression analysis of this dataset shows that weight is significantly correlated with the prevalence of *Staphylococcus* and *Enterobacteriaceae* (% *Enterobacteriaceae* - % *Staphylococcus*) but that this prevalence is not linked with postconceptional age (Fig. 5a). A Mixed Effect Regression analysis was subsequently performed to take the longitudinal and paired aspect of this sample-set into account (multiple samples per infant over time) and came to the same conclusions (Fig. 5b-d); the abundance of *Staphylococcus* and *Enterobacteriaceae* are associated with weight (*p* = 0.01, both) but not with postconceptional age in this dataset.

### Compositional nature microbiome data

Microbiome data is compositional and as such does not take into account any differences in the levels of microbial biomass that exists between samples. A clear example of this can be found when analyzing the first meconium samples in regards to the day of obtaining such a first meconium sample when looking at reagent contamination levels. Directly after delivery meconium samples will be largely devoid of microbial biomass. You might be able to detect some initial colonizers, like those derived from the mode of delivery, but these initial colonizers will not have proliferated much making it dubious whether we’re dealing with microbiomes where competition between species (based upon suitability) plays a large role yet. As the amount of bacterial DNA derived from samples from the earliest days will be lower there will be more room for reagent contaminants to be amplified, sequenced and measured. Resultingly, when looking at samples obtained from the first meconium there is a significant negative correlation between the day of obtaining the first meconium after delivery and the % of reagent contamination reads (rho = 0.4, *p* = 0.01) as the level of microbial biomass increases steadily.

Changes in microbiome composition might thus for example not be due to one group of bacteria becoming less prevalent (*Staphylococcus*) as another replaces it (*Enterobacteriaceae*) as a result of competition, but simply be the result of e.g. *Enterobacteriaceae* becoming much more prevalent on its own irrespective of *Staphylococcus* levels (increased microbial biomass). To circumvent bias in comparisons as the result of differences in microbial load one should instead look at the ratio’s of the main bacterial groups of interest [33]. The most important pattern of note observed in these analyses is the apparent shift from a microbiome dominated by *Staphylococcus* towards one dominated by *Enterobacteriaceae* as infants gain weight/mature. The log value of the *Enterobacteriaceae*: *Staphylococcus* ratio is found to be positively correlated with weight (rho = 0.31, *p* < 0.01) whilst postconceptional age is not (rho=-0.04, *p* = 0.60). By excluding meconium samples which are extremely low biomass (where competition is questionable) the association of this ratio with weight becomes even stronger (rho = 0.38, *p* = < 0.01 whilst the association with age remains absent (rho = 0.05, *p* = 0.64).

### Intestinal microbiota development and health

While *Staphylococcus* was associated with low absolute weight in this cohort, they did not appear detrimental to health as (1) the amount of weight gain during any single week did not correlate with the gut microbiome composition (or any of the individual species) at the start of that week and (2) their previously reported negative association with necrotizing enterocolitis development was similarly found within this cohort in meconium samples (*p* = 0.034). Overall infant mortality in this cohort, in part caused by necrotizing enterocolitis (*n* = 8 in total; 3 caused by NEC), was not aligned with the gut microbiome composition or with BW (*p* = 0.36) but it was negatively associated with GA at birth (*p* = 0.005) and positively but not significantly (*p* = 0.08) with prolonged premature rupture of membranes (PPROM).

### Exogenous factors and microbiota development

Exogenous factors such as antibiotics use and/or feeding regime were found to be of ancillary importance in comparison with patterns associated with the mode of delivery or with absolute body weight. Associations of bacterial groups with antibiotics use were either found to be non-significant or disappeared when adjusting the analyses for mode of delivery. The current number of subjects was insufficient to unravel significant associations between microbiome development with antibiotics and feeding regimes.

## Discussion

This study, which prospectively investigated the development of the microbiome of extremely preterm infants during the first four weeks of life, has two main findings. First, confirming current data [34], differences caused by mode of delivery have a strong but transient influence on intestinal microbiome composition. Importantly, this effect can already be observed in the meconium. Whilst meconium is formed inside the womb, we consider it unlikely that bacteria colonized the meconium in utero (before delivery); bacterial colonizers most likely, as the mode of delivery effects shows, quickly get into the meconium during and after delivery. Exceptions to this might however be expected in cases of PPROM and/or uterine infections. We however did not see strong evidence for this; the effect of mode of delivery far exceeds this potential effect. Furthermore, the amount of microbiala biomass is typically much lower in meconium samples than in subsequent samples as reagent contamination analysis show. Our results are in contrast to findings of others [35] who perhaps do not correct for reagent contamination in such low biomass samples [36].

Second, this study reveals that current absolute weight is a better marker for the maturation of the infants’ intestinal microbiome than postconceptional age as it less confounded by various infant-specific factors. Our results support the hypothesis that the intestinal microbiome of the preterm infant appears to follow a patterned progression which is indicative of the microbiome developing as a result of infant development measured in weight, not the weight developing as a result of the microbiome. We did not find that the microbiome composition at any taxonomic level in paired analyses was predictive for weight gain in subsequent weeks. Important in this regard is to again note that bifidobacteria or lactobacilli are generally absent in extremely premature children. The abundance, or the diminished abundance in the case of antibiotics [37], of these bacteria is frequently linked with improved growth. Similarly, the use of probiotics in term and preterm infants has been found associated with improved weight gain [38]. Bifidobacteria and lactobacilli have co-evolved together with humans in a mutually beneficial relationship. The same cannot be said for bacteria associated with an extremely preterm microbiome as preterm infants would simply have died in the past; (extremely) preterm infants represent an ecologically unnatural situation where bifidobacteria and lactobacilli have trouble even persisting without continually being supplied in relatively large quantities as probiotics.

Recently, La Rosa et al. [26] and Korpela et al. [39] both described the hypothesis that the gut microbiome of the preterm infant appears to follow a patterned progression linked with postconceptional age as a key marker for host biology / maturity. La Rosa, et al. [26] describes a progression from bacilli to γ-Proteobacteria (*Enterobacteriaceae*) to clostridia (and Negativicutes). In this hypothesis, the place where preterm infants step into this progression is mainly dependent on their GA at birth [26]. A longer follow-up study would have seen a subsequent progression into *Bifidobacterium* (and/or *Bacteroides*). Our study, which focused on the first 4 weeks of life of extremely and very preterm infants, confirms the first part of this transition, namely the progression of a staphylococci (bacilli) dominance to an *Enterobacteriaceae* dominated microbiome in extremely preterm infants. We however found in this dataset that postconceptional age was not a meaningful descriptor of maturity/host biology with regard to the development of the gut microbiome as various complications can hamper infant development which are not taken into account by age yet which are represented by weight. A reason for this inaccuracy is highlighted by our comparison of infants delivered vaginally or by CS and the underlying causes of prematurity. In our cohort CS delivered infants had on average a higher GA at birth but a lower BW (Z-score) than their vaginally delivered counterparts (Fig. 1). As can be expected, we observed that the type of pregnancy complication inducing preterm birth (placental insufficiency vs. intra-uterine infection/spontaneous preterm birth) was not only significantly associated with the mode of delivery but also with Z-scores for BW, representing fetal growth restriction.

In a cohort observed by Ho et al. [16] they similarly found that bacilli and *Enterobacteriaceae* formed the dominant groups but they ascribed their findings to a dichotomous development of the gut microbiome. The dichotomous development of the gut includes one cluster (I) of samples starting off with a high abundance of staphylococci which in time gave way to an increased abundance of *Enterobacteriaceae* and the other cluster (II) starting off with a high abundance of *Enterobacteriaceae* that declined slowly as clostridia became more prominent [16]. The developments in these two clusters fit perfectly into the aforementioned patterned progression if absolute weight is used as a marker of intestinal microbiome maturity instead of postconceptional age. Instead of a threshold for GA, which La Rosa [26] suspected, there might be a weight-determined threshold, which influences the gut microbiome maturation. In the cohort of Ho et al., infants from cluster I and II had a similar GA at birth on average (28.0 vs. 27.9) but infants assigned to cluster I had a BW of 1053 g while those assigned to cluster II had a BW of 1176 g [16]. In our study we found that the switch (threshold) between staphylococci and *Enterobacteriaceae* was particularly evident around 1100 g (Fig. 5), consistent with the difference between these 2 clusters.

The main driver for this difference (weight vs. postconceptional age) in this study is the cause of prematurity; placental insufficiency was typically accompanied by CS and was logically negatively associated with lower BW Z-scores due to fetal growth restriction of the infant. Furthermore, some infants thrive and concomitantly gain weight after birth while others do not, although both increase equally in gestational/postnatal age. We observed that absolute weight, as a logical key marker for host biology, does not suffer from these confounders and could be used to visualize the patterned progression of staphylococci (bacilli) to *Enterobacteriaceae* (Fig. 5) in this dataset. The underlying mechanism why weight influences the gut microbiome maturation is not understood. We hypothesize that fetal growth restriction / low (birth) weight influences immune response modulation and that altered intestinal development (i.e. influencing maturation of paneth cells, mucus production) could cause weight dependent microbiome maturation differences [40].

Exogenous factors other than the mode of delivery such as antibiotics, neonatal feeding regime were also analyzed with regard to their association with the intestinal microbiome but no significant correlations were found in this cohort. While such external factors have been found to be associated with certain aspects of the gut microbiome development, they do not appear to represent the main drivers of gut maturation in preterm infants. More samples would be required to study these factors. Maternal data, such as the results of vaginal swabs and/or the use of maternal antibiotics, were not available for this study but could be of possible relevance for initial colonization.

This study highlights that the intestinal gut microbiome development in extremely and very preterm infants is mainly driven by absolute weight but is initially strongly affected by the mode of delivery. It also stresses the importance of pregnancy complications with diminished placental function as it directly affects the actual physical maturity level of the infant in which in turn directly affects the stage of progression [26, 39] from which the gut microbiome development starts. Underlying mechanisms behind possible weight thresholds are not yet understood. There is a need for more knowledge on the affect of (birth) weight on immunological responses and organ maturation. Before interventions are implemented, such as targeted antibiotic therapies or the use of pre- and probiotics, it is critical to understand which organisms are to be considered ‘normal’ (part of the maturation process), in regards to the level of gut development of the infant at a certain weight, and which ones are indicative of potential dysbiosis/disease. For example, *K. oxytoca* or *K. pneumonia*, which belong to the family *Enterobacteriaceae*, have been frequently associated with, amongst other things, sepsis in newborns, but they appear to be a ‘normal’ part of the patterned progression of the bacteria composition as the infant gut matures [16, 26]. Trials with i.a. strain resolved metagenomic analyses of a larger number of samples might show that only particular *Klebsiella* strains are to be associated with disease directly or indirectly. Colonization with *Klebsiella* appears to be normal whereas sepsis with *Klebsiella* might merely be a symptom of other things going wrong in the preterm infants’ gut [41, 42]. Our results underline the importance of larger multi-center observational studies to reveal the exact intestinal microbiome maturation of the extremely preterm infant and its underlying driving factors, such as immunomodulation.

The main limitation of this study is that sample numbers are still relatively limited leading to insufficient power to say anything for example about exogenous factors such as antibiotics use and/or feeding regime. To retain as much power and sensitivity as possible we did not exclude samples with a low but still adequate sequencing depth nor did we rarify. Alpha or beta diversity analyses, which can be affected by differences by in sequencing depth [43], were not utilized in this study and an increased potential for a higher false discovery rate was countereacted by focusing on the compositionaly important bacterial groups which due to their quantity (%) are effectively unaffected by a relatively low sequencing (> 2000 reads) depth.

## Conclusions

During the first four weeks of life the gut microbiome of extremely and very preterm infants (birth weight < 1000 g) undergoes, or starts to undergo, a transition from a gut microbiome with a high abundance of staphylococci towards one dominated by *Enterobacteriaceae*. This study improves the patterned progression hypothesis [26] by using the infants’ absolute weight at sampling time as a more accurate marker of host biology and maturation than postconceptional age. These findings were independent of pregnancy complications with diminished placental function. We hypothesize that weight thresholds determine the stage of the progression in maturation and development of the colonization process. Exogenous factors were in this cohort, apart from the strong yet transient effect of the mode of delivery, of minor importance.

## Materials and methods

### Subjects

This study is part of a prospective observational trial (CALIFORNIA trial; registered as NTR4153 in the Dutch trial registry, and approved by the Medical Ethics Committee of the University Medical Center Groningen), which studied infants for developing NEC [5, 6, 44–47]. We confirm that all methods were performed in accordance with the guidelines and regulations, including the declaration of Helsinki. The CALIFORNIA trial included 100 infants admitted to the Neonatal Intensive Care Unit of the University Medical Center Groningen between October 2012 and December 2014, after written informed consent of their parents was given. Infants born at a GA of ≤ 30 weeks and born with a BW of ≤ 1000 g were applicable for the study from whom more than two samples during the first 4 weeks were available. Patients with other abdominal diseases (abdominal wall defects or congenital intestinal atresia) were excluded from this trial. Patient characteristics and demographic variables were derived from the CALIFORNIA database.

### Demographic and clinical variables

Data from each sample day were used. Variables consisted of mode of delivery, BW, GA at birth, z-score BW (which represents the standard deviation in SD units from the Dutch reference growth curves) [48], bodyweight on the sample day, the administration of mothers’ milk and / or of formula milk in milliliters/kg on the sample day, the antibiotic use on the sample day and if antibiotics were administered for more than 48 h after birth. Complications during pregnancy were classified as placental insufficiency (pre-eclampsia/HELLP, fetal growth restriction and fetal distress) and intra-uterine infection/spontaneous preterm birth (chorioamnionitis, PPROM, cervical insufficiency and premature contractions).

### Faecal samples

We intended to analyze one faecal sample per week, starting with the first meconium, and afterwards the first faecal sample of every week during the first four weeks after birth. Faecal samples were stored at – 80 °C prior to the start of this study. The first sample of each week was used for analyses of the current sub-study.

### DNA extraction and sequence library preparation

Faecal DNA was extracted from a 0.25 g faecal sample by double bead beating in combination with the QIAamp DNA Mini kit (Qiagen; Hilden, Germany), as described by a study that used the same technique [49]. Polymerase chain reaction (PCR) amplification targeted the V3 and V4 region of the 16 S rRNA gene by using modified 341 F and 806R primers [50, 51]. The 806R primers contained a 6-nucleotide barcode. An elaborated explanation of the PCR reaction, DNA cleanup, and MiSeq library preparation is found in the Supplemental data file 1 and Supplementary Table 1.

### Analyses of sequence reads

The 16 S rRNA amplicon data was processed using MOTHUR [52] and the Oligotyping pipeline [53] as described by de Goffau et al. [54] with the following exceptions. All fastq files from all samples (from 3 separate MiSeq runs) were processed together in one batch instead of in parallel as described previously [54]. Assembled contigs from the MOTHUR pipeline were similarly processed with PRINSEQ-lite [55] but only to remove the four poly NNNNs present in the forward adaptor/prime using the ‘-trim_left 4’ command. Similarly, the PRINSEQ trimmed sequences were used for the first ‘screen.seqs’ command to remove ambiguous sequences and sequences containing homopolymers longer than 6 bp. In addition, any sequences longer than 5000 bp or shorter than 400 bp were removed. Unique reads (‘unique.seqs’) were aligned (‘align.seqs’) using the Silva bacterial database ‘silva.nr_v138.align’ with flip parameter set to true. Any sequences outside the expected alignment coordinates were removed. The correctly aligned sequences were subsequently filtered (‘filter.seqs’) with ‘vertical = T’ and ‘trump=’. The filtered sequences were de-noised by allowing three mismatches in the “pre.clustering” step and chimaeras were removed using Uchime with the dereplicate option set to ‘true’. The chimaera-free sequences were classified using the Silva reference database ‘silva.nr_v138.align’ and the Silva taxonomy database ‘silva.nr_v138.tax’ and a cut-off value of 80 %. Chloroplast, mitochondria, unknown, and eukaryota sequences were removed. All reads from each sample were subsequently renamed, placing the sample name of each read in front of the read name. The last MOTHUR step was the ‘deunique.seqs’ command, which creates a redundant fasta file from a fasta and name file. After the MOTHUR pipeline, the redundant fasta file, which now only contains high-quality aligned fasta reads, was subsequently used for oligotyping using the unsupervised minimum entropy decomposition (MED) for sensitive partitioning of high-throughput marker gene sequences [53]. A minimum substantive abundance of an oligotype (-M) was defined at 100 reads and a maximum variation allowed (-V) was set at 3 using the command line ‘decompose redundant.fasta -M 100 -V 3 -g –t’. The node representative sequence of each oligotype (OTP) was used for species profiling using the ARB program (v.5.5-org-9167) [56] extracting the top 2 hits. The species identification with ARB were subsequently double-checked with BLAST using the NCBI online BLAST interface (blastn, database: Others, exclude: uncultured) [57]. Nearly always one or both of the 2 top hits of ARB were consistent with BLAST results. Supplementary Information File 2 provides detailed information on all bacterial groups, including representative aligned sequences, sequencing run information, reagent contamination removal & abundances per bacterial group per sample, and extensive metadata per sample providing direct access to all the relevant data.

As meconium samples in particular, but also many later samples have low bacterial biomasses, reagent contamination needs to be accounted for. Reagent contamination recognition analyses were subsequently performed as described by de Goffau et al. [49], using the Spearman’s rank correlation coefficients method. The consistency of the ratio of reagent-derived species within samples allows for their rapid identification. As a result, all reads from oligotypes identified as *Undibacterium oligocarboniphilum*, *Acinetobacter guillouiae*/*iwoffii*, *Curvibacter lanceolatus*, *Sphingomonas echinoides*, *Ralstonia pickettii, Delftia acidovorans*, *Ralstonia insidiosa*, *Sphingomonas kyeonggiensis*, *Methylorubrum extorquens*, *Phyllobacterium myrsinacearum*, *Sphingomonas panni*, *Sphingomonas faeni*, *Sediminibacterium salmoneum*, *Rhodococcus erythropolis* and *Pelomonas saccharophila* were removed before further analysis. The number of reads per sample before reagent contamination removal had an interquartile range of 35,190–58,046 (median: 48,414) with 5 outliers above 100,000 reads and similarly 5 outliers below 10,000 reads (minimum: 2145). After reagent contamination removal the interquartile range was 33,968–57,180 (median: 45,597) with 5 outliers above 100,000 reads and 9 samples below 10,000 reads (minimum: 913). Analyses strongly influenced by sequencing depth (like diversity analyses) were not performed in this study; only relative abundances of the most abundant microbial groups are analyzed in detail.

### Statistics

Statistical analyses were conducted with IBM SPSS Statistics 21.0, Past3.14 [58], PRISM 8 (v8.3.1) and the Calypso web portal (version 8.84) [59]. Combinations of principal component analyses (PCA), regression and paired analyses were performed to examine the relationship between the microbiota and the following factors: mode of delivery, the birth weight, gestational age at birth, z-score birth weight, bodyweight on the sample day, the administration of mothers’ milk and / or of formula milk in milliliters/kg on the sample day, the antibiotic use on the sample day and antibiotic use for more than 48 h after birth. Two sided P-values less than 0.05 were considered statistically significant. Unless otherwise indicated, the Mann-Whitney-U test or Chi-square test were used to test differences between groups. Testing of the correlation between parameters was done with the Spearman’s correlation test, while one-way-ANOVA was used to assess individual parameters development in time. A Mixed Effect Regression analysis was performed to take the longitudinal and paired aspect of this sample-set into account (multiple samples per infant over time) for analyzing the relationship between the gut microbiota composition maturation measured either via gestational/postnatal age or the actual infant weight as each sample was taken. The option for Mixed Effect Regression was used from the “Univariate” drop down menu from Calypso web portal using total sum scaling (TSS) normalized i.e. relative abundance data. The default data transformation was disabled. Paired analysis were furthermore performed to correlate weight gain between consecutive weeks (Weight_n_ – Weight_n+1_), in an infant-specific paired fashion, with the abundance of bacterial groups at the start of each week. The 4 timepoints resulted in 3 of such analyses and we performed these analyses on the OTU level, the genus level and the family level yet we did not find a single significant association (or even something resembling a trend) in any of these 3 analyses at any phylogenetic level. Microbial composition did not appear to be predictive in regards to weight gain.

## Supplementary Information

**Additional file 1.**

**Additional file 2.**

**Additional file 3.**

## Data Availability

The datasets generated and analysed during the current study are avaible on request. The sequencing data is available at BioProject ID: PRJNA729760. Supplementary Information File 2 provides detailed information on all bacterial groups, including representative aligned sequences, sequencing run information, reagent contamination removal & abundances per bacterial group per sample & extensive metadata per sample providing direct access to all the relevant data.

## Abbreviations

BW: Birth weight
CS: Caesarean section
DNA: Deoxyribonucleic acid
GA: Gestational age
HELLP: Hemolysis, Elevated Liver enzymes, Low platelets
NEC: Necrotizing enterocolitis
NICU: Neonatal intensive care unit
OTP: Oligotype
PCR: Polymerase chain reaction
PPROM: Preterm premature rupture of membranes
RNA: Ribonucleic acid
