# Supplementary material for: Weight shapes the intestinal microbiome in preterm infants: results of a prospective observational study
Source: BMC Microbiol. 2021 Jul 21;21:219. doi: 10.1186/s12866-021-02279-y (PMC8293572; doi:10.1186/s12866-021-02279-y)
Supplement: Supplementary file 1 — Additional file 1. [file 12866_2021_2279_MOESM1_ESM.docx]

**Supplemental Data file 1: PCR & MiSeq preparation**

Reaction conditions consisted of an initial 94 ^o^C for 3 min followed by 32 cycles of 94 ^o^C for 45 sec, 50 ^o^C for 60 sec, and 72 ^o^C for 90 sec, and a final extension of 72 ^o^C for 10 min. An agarose gel confirmed the presence of product (band at ~465 base pairs) in successfully amplified samples. The remainder of the PCR product (~45 μl) of each sample was mixed thoroughly with 25 μl Agencourt AMPure XP magnetic beads and were incubated at room temperature for 5 minutes. Beads were subsequently separated from the solution by placing the tubes in a magnetic bead separator for 2 minutes. After discarding the cleared solution the beads were washed twice by resuspending the beads in 200 μl freshly prepared 80% ethanol, incubating the tubes for 30s in the magnetic bead separator and and subsequently discarding the cleared solution. The pellet was subsequently air dried for 15 minutes and resuspended in 52.5 μl 10 mM Tris HCl pH 8.5 buffer. 50 μl of the cleared up solution is subsequently transferred to a new tube. The DNA concentration of each sample was done using a Qubit® 2.0 fluorometer (www.invitrogen.com/qubit) and the remainder of the sample was stored at -20^o^C until library normalization ^o^C. Library normalization was done the day before running samples on the MiSeq by making 2 nM dilutions of each sample. Samples were pooled together by combining 5 μl of each diluted sample. 10 μl of the sample pool and 10 μl 0.2 M NaOH were subsequently combined and incubated for 5 minutes to denature the sample DNA. To this, 980 μl of the HT1 buffer from the MiSeq 2x300 kit is was subsequently added. A denatured diluted PhiX solution was made by combining 2 μl of a 10 nM PhiX library with 3 μl 10 mM Tris HCl pH 8.5 buffer with 0.1% Tween 20. These 5 μl were mixed with 5 μl 0.2 M NaOH and incubated for 5 minutes at room temperature. These 10 μl were subsequently mixed with 990 μl HT1 buffer. 150 μl of the diluted sample pool was combined with 50 μl of the diluted PhiX solution and is further diluted by adding 800 μl HT1 buffer. 600 μl of the prepared library was loaded into the sample loading reservoir of the MiSeq 2x300 cartridge.
