# Supplementary material for: Weight shapes the intestinal microbiome in preterm infants: results of a prospective observational study
Source: BMC Microbiol. 2021 Jul 21;21:219. doi: 10.1186/s12866-021-02279-y (PMC8293572; doi:10.1186/s12866-021-02279-y)
Supplement: Supplementary file 3 — Additional file 3. [file 12866_2021_2279_MOESM3_ESM.docx]

**Supplemental Table S1B**

| V3_F_modifi ed | aatgatacggcgaccaccgagatctacactctttccctacacgacgctcttccgatctNNNNCCTACGGGAGGCAGC AG |
| --- | --- |
| V4_1R | caagcagaagacggcatacgagatCGTGATgtgactggagttcagacgtgtgctcttccgatctGGACTACHVGGG TWTCTAAT |
| V4_2R | caagcagaagacggcatacgagatACATCGgtgactggagttcagacgtgtgctcttccgatctGGACTACHVGGG TWTCTAAT |
| V4_3R | caagcagaagacggcatacgagatGCCTAAgtgactggagttcagacgtgtgctcttccgatctGGACTACHVGGG TWTCTAAT |
| V4_4R | caagcagaagacggcatacgagatTGGTCAgtgactggagttcagacgtgtgctcttccgatctGGACTACHVGGG TWTCTAAT |
| V4_5R | caagcagaagacggcatacgagatCACTGTgtgactggagttcagacgtgtgctcttccgatctGGACTACHVGGG TWTCTAAT |
| V4_6R | caagcagaagacggcatacgagatATTGGCgtgactggagttcagacgtgtgctcttccgatctGGACTACHVGGG TWTCTAAT |
| V4_7R | caagcagaagacggcatacgagatGATCTGgtgactggagttcagacgtgtgctcttccgatctGGACTACHVGGG TWTCTAAT |
| V4_8R | caagcagaagacggcatacgagatTCAAGTgtgactggagttcagacgtgtgctcttccgatctGGACTACHVGGG TWTCTAAT |
| V4_9R | caagcagaagacggcatacgagatCTGATCgtgactggagttcagacgtgtgctcttccgatctGGACTACHVGGG TWTCTAAT |
| V4_10R | caagcagaagacggcatacgagatAAGCTAgtgactggagttcagacgtgtgctcttccgatctGGACTACHVGGG TWTCTAAT |
| V4_11R | caagcagaagacggcatacgagatGTAGCCgtgactggagttcagacgtgtgctcttccgatctGGACTACHVGGG TWTCTAAT |
| V4_12R | caagcagaagacggcatacgagatTACAAGgtgactggagttcagacgtgtgctcttccgatctGGACTACHVGGG TWTCTAAT |
| V4_13R | caagcagaagacggcatacgagatCGTACTgtgactggagttcagacgtgtgctcttccgatctGGACTACHVGGG TWTCTAAT |
| V4_14R | caagcagaagacggcatacgagatGACTGAgtgactggagttcagacgtgtgctcttccgatctGGACTACHVGGG TWTCTAAT |
| V4_15R | caagcagaagacggcatacgagatGCTCAAgtgactggagttcagacgtgtgctcttccgatctGGACTACHVGGG TWTCTAAT |
| V4_16R | caagcagaagacggcatacgagatTCGCTTgtgactggagttcagacgtgtgctcttccgatctGGACTACHVGGG TWTCTAAT |

Nucleotide sequences of primers used in the construction of libraries for Illumina sequencing. Lowercase letters denote adapter sequences necessary for binding to the flowcell, underlined lowercase are binding sites for the Illumina sequencing primers, bold uppercase highlight the index sequences (additional indexes are described in the paper by Bartram et al.). Regular uppercase are the V3 and V4 region primers (341F on for the forward primer and 806R for the reverse primers). The inclusion of four maximally degenerated bases (“NNNN”) maximizes diversity during the first four bases of the run. Diversity is important for identifying unique clusters and base-calling accuracy [11, 12].

References

- Bokulich NA, Joseph CM, Allen G, Benson AK, Mills DA (2012) Next-generation sequencing reveals significant bacterial diversity of botrytized wine. PLoS One 7:e36357, DOI:10.1371/journal.pone.0036357

- Bartram AK, Lynch MD, Stearns JC, Moreno-Hagelsieb G, Neufeld JD (2011) Generation of multimillion-sequence 16S rRNA gene libraries from complex microbial communities by assembling paired-end illumina reads. Appl Environ Microbiol 77:3846-3852, DOI:10.1128/AEM.02772-10
